# Characterization of the *algC* Gene Expression Pattern in the Multidrug Resistant *Acinetobacter baumannii* AIIMS 7 and Correlation with Biofilm Development on Abiotic Surface

**DOI:** 10.1155/2014/593546

**Published:** 2014-12-03

**Authors:** Praveen K. Sahu, Pavithra S. Iyer, Sagar H. Barage, Kailas D. Sonawane, Balu A. Chopade

**Affiliations:** ^1^Institute of Bioinformatics and Biotechnology, University of Pune, Pune 411 007, India; ^2^Ispat General Hospital, SAIL, Rourkela 769 005, India; ^3^Department of Biotechnology, Shivaji University, Kolhapur 416 004, India; ^4^Structural Bioinformatics Unit, Department of Biochemistry, Shivaji University, Kolhapur 416 004, India; ^5^Dr. Babasaheb Ambedkar Marathwada University, Aurangabad 431 001, India

## Abstract

Relative quantification of *algC* gene expression was evaluated in the multidrug resistant strain *Acinetobacter baumannii* AIIMS 7 biofilm (3 to 96 h, on polystyrene surface) compared to the planktonic counterparts. Comparison revealed differential *algC* expression pattern with maximum 81.59-fold increase in biofilm cells versus 3.24-fold in planktonic cells (*P* < 0.05). Expression levels strongly correlated with specific biofilm stages (scale of 3 to 96 h), coinciding maximum at initial surface attachment stage (9 h) and biofilm maturation stage (48 h). Cloning, heterologous expression, and bioinformatics analyses indicated *algC* gene product as the bifunctional enzyme phosphomannomutase/phosphoglucomutase (PMM/PGM) of ∼53 kDa size, which augmented biofilms significantly in *algC* clones compared to controls (lacking *algC* gene), further localized by scanning electron microscopy. Moreover, molecular dynamics analysis on the three-dimensional structure of PMM/PGM (simulated up to 10 ns) revealed enzyme structure as stable and similar to that in *P. aeruginosa* (synthesis of alginate and lipopolysaccharide core) and involved in constitution of biofilm EPS (extracellular polymeric substances). Our observation on differential expression pattern of *algC* having strong correlation with important biofilm stages, scanning electron-microscopic evidence of biofilm augmentation taken together with predictive enzyme functions via molecular dynamic (MD) simulation, proposes a new basis of *A. baumannii* AIIMS 7 biofilm development on inanimate surfaces.

## 1. Introduction

In recent years,* Acinetobacter baumannii* has been listed as one of the most important nosocomial pathogens [1–3]. The pathogen has become a universal challenge to treatment, owing to its multidrug resistant (MDR) nature and a plethora of virulence attributes [2, 4, 5]. Associated mortality up to 30% is seen with* A. baumannii* infections [6], such as ventilator-associated pneumonia, bacteraemia, urinary tract infections, burn wound infections, endocarditis, secondary meningitis, and septicemia especially in intensive care units [1].* A. baumannii* infection and colonization often involve biofilm formation [7] on either abiotic [8, 9] or biotic surfaces [10, 11]. Biofilm formation is a virulence trait in* A. baumannii* which is of multifactorial nature [4, 12]. The process of biofilm development in* A. baumannii* is a highly regulated process and could be the interplay of several genetic determinants [13]. The extracellular matrices of bacterial biofilm comprise of proteins, nucleic acids, and polysaccharides [14] which are often considered as ideal start-points to further investigation towards effective treatment measures against biofilm-associated pathogens, such as MDR* A. baumannii*.

*Acinetobacter* spp. and* Pseudomonas aeruginosa* together are known to be responsible for a significant proportion of nosocomial infections [15] with crude mortality rates of 30% to 75% in case of nosocomial pneumonia only [16]. In patients with cystic fibrosis, alginate production by* P. aeruginosa* is found to be associated with high morbidity and mortality [17, 18]. Production of alginate, an exopolysaccharide, is responsible for development of mucoid bacterial phenotype [17, 19] which is associated with biofilm formation in* P. aeruginosa* under iron limiting conditions [20]; although proportions of alginate in the extracellular polysaccharide/polymeric substances (EPS) of biofilm could vary significantly in strains like PA14 and PA01 of* P. aeruginosa* [21]. It acts as an intercellular material in complex biofilm structures and facilitates nonspecific attachment of bacteria to surfaces, thus increasing cohesion [22]. Biosynthesis of alginate is well characterized in* P. aeruginosa* [23, 24] and* Escherichia coli* [25]. The gene* algC* in* P. aeruginosa* codes for a crucial bifunctional enzyme phosphomannomutase/phosphoglucomutase (PMM/PGM), belonging to the *α*-D-hexomutase superfamily, synthesizing alginate and lipopolysaccharide (LPS) core, respectively [24, 26, 27]. The genetic regulation of* algC* gene in* P. aeruginosa* [23, 24, 28] could be dependent on surface attachment and other important factors.

Earlier study shows biofilm formation by clinical strains* A. baumannii* on abiotic surface (urinary catheters) as a major reason for device-related infections [29]. To explore the basis of persistence on such clinically important (abiotic) surfaces, we characterized the role of extracellular macromolecules like extracellular DNA (eDNA), a major component of the biofilm EPS matrix and evaluated its role in biofilm formation [9]. Besides eDNA, other extracellular macromolecules such as exopolysaccharides also play significant role in the constitution of the biofilm EPS. Recent findings on the EPS of* A. baumannii* indicate it as a universal protector from antibiotics like tobramycin [30] and one of its capsular polysaccharides (K1) being highly immunogenic and a potential therapeutic target via passive immunization [31]. The synthesis of specific exopolysaccharides like alginate and LPS core is well characterized in* P. aeruginosa* along with their involvement in biofilm formation as mentioned earlier; however, remains unclear in* A. baumannii*, which is a close relative and a globally important nosocomial pathogen. Presumably, the genetic basis of the association of exopolysaccharides such as alginate and LPS core with biofilm formation may exacerbate* A. baumannii* infections on clinically important surface, which can multiply the intrinsic antibiotic resistance and worsen the treatment scenario. However, there are no reports as yet, which describe the genetic association of* algC* with biofilm formation in* A. baumannii*. Therefore in this study, we set out to characterize the* A. baumannii algC* gene expression pattern during biofilm formation and its association with the biofilm development, which could further pave way for future research on potential drug target(s) for biofilm-associated infections caused by MDR* A. baumannii*.

In the current study, we identified the* algC* gene in the MDR strain of* A. baumannii* AIIMS 7 genome and characterized its quantitative gene expression patterns in growing biofilm cells compared to the planktonic cells using the relative quantification (ΔΔCt method) in real-time PCR. Gene expression pattern was correlated with specific stages of biofilm development. Molecular dynamics (MD) simulation was performed on the three dimensional (3D) structure of the gene product (enzyme PMM/PGM) to confirm the stability and to compare with that of* P. aeruginosa*, besides phylogenetic analysis. Subsequently, biofilm augmentation due to* algC* gene was evaluated using cloning and heterologous expression studies followed by scanning electron microscopy.

## 2. Materials and Methods

### 2.1. Bacterial Strains and Culture Conditions

Multidrug resistant clinical isolate of* Acinetobacter baumannii* (strain AIIMS 7) was used in this study, as described previously [9]. The bacterium was grown on CLED (cysteine-lactose electrolyte-deficient) agar and Luria Bertani (LB) broth (HiMedia, India).* E. coli* DH5*α* was used for the cloning and heterologous expression experiments. Clones were maintained on Luria agar plates containing 100 mg/L ampicillin.

### 2.2. Nucleic Acids Purification and PCR

Genomic DNA was purified using a DNA isolation kit (Sigma Aldrich, USA). Total RNA and plasmid purification was done using Trizol reagent (Invitrogen, USA) and GenElute plasmid extraction kit (Sigma Aldrich, USA) respectively, according to manufacturer's instructions. Concentration and purity criteria of the nucleic acids were quantified in a BioPhotometer Plus (Eppendorf, Germany). Primers used for amplification of direct PCR for* algC* and RT-PCR of internal regions of* algC* are listed in Table 1. Primers were designed using Primer Quest (IDT, USA) and synthesized (Sigma Aldrich, USA). For amplification of the 1781 bp fragment* algC* from genomic DNA, PCR conditions used were initial denaturation of 5 min at 94°C, followed by 30 cycles of 30 sec at 94°C, 20 seconds at 54.5°C, and 45 seconds at 72°C with final extension of 5 minutes at 72°C. Amplification of* algC* was also verified with native plasmids as PCR template. PCR assays were performed in an ep-gradient PCR (Eppendorf, Germany); products were separated on agarose gels stained with ethidium bromide and documented in a gel documentation system (Alpha Imager HP, Alpha Innotech, USA). DNase-, RNase-, Protease-free water was used as negative control. All PCR chemicals and reagents were purchased from Sigma unless stated otherwise.

### 2.3. Confirmation of Internal Regions of cDNA and DNA Sequencing

To confirm the transcription of* algC*, two internal regions of* algC* cDNA (1360 bp and 463 bp) were amplified using upstream primer 363F and CD1701R and internal nested primers 1260 nest1F and CD1701R, respectively (Table 1). Total RNA samples were treated with DNase I and then used in RT-PCR, which was performed using a single-step Reverse Transcription kit (Promega, USA). To test possible DNA contamination in RNA samples, direct PCR of total RNA without reverse transcription was performed. DNase-, RNase-, Protease-free water was used as negative control. RT-PCR was performed with one *μ*L of cDNA template and 100 pm of primers. cDNA from* E. coli* DH5*α* with pGEM-3Zf (+) plasmid as empty vector (Applied Biosystem, USA) served as negative control. DNA sequencing was performed in the Applied Biosystems 3730 DNA Analyzer platform. Sequences were analyzed by sequence analysis software v5.1.1 (Applied Biosystems, USA) and assembled using software ContigExpress (Vector NTI Advance v11.5.0, Invitrogen). Promoter and the ribosome binding sites of* algC* were predicted using online tool Neural Network Promoter Prediction program (http://www.fruitfly.org/seq_tools/promoter.html).

### 2.4. Total RNA Extraction and cDNA Synthesis from Biofilm and Planktonic* A. baumannii* Cells

To compare gene expression pattern of* algC* in biofilm versus planktonic* A. baumannii* AIIMS 7, total RNA was extracted from cells growing in biofilm and planktonic mode and then real-time quantitative RT-PCR was performed. Biofilms were allowed to form in 6-well polystyrene plates (Tarsons, India) beginning at 3 hours up to 96 hours.* A. baumannii* AIIMS 7 cultures (10^6^ CFU/mL, overnight grown) were inoculated onto the wells and diluted initially with sterile distilled water (without LB broth) at a dilution of 1 : 40. After incubation for 3 hours at 37°C under static conditions, the supernatant was discarded and fresh sterile nutrient medium (Luria broth) was added at a final dilution of 1 : 40 and then incubated under similar conditions to allow biofilm formation. After appropriate incubation(s), plates were taken out; nonadherent cells were aspirated and discarded after brief sonication. Plates containing biofilms were washed twice with phosphate buffer saline (PBS). Surface-attached bacteria (biofilm forming) were scraped off and total RNA was purified using Trizol reagent (Invitrogen, USA) as per manufacturer's instructions. Similarly, total RNA was purified from planktonic cell culture at the corresponding time points (3–96 h). RNA samples were treated with DNase I (Sigma, USA). To test possible DNA contamination in RNA samples, direct PCR of total RNA without reverse transcription was done. cDNA was synthesized using Verso cDNA synthesis kit (Thermo, USA) as per manufacturer's instructions. cDNA concentrations were determined using BioPhotometer Plus (Eppendorf, Germany).

### 2.5. Relative Quantification of* algC* Expression Pattern by Real-Time PCR

Specific primers and probes for* algC* gene along with* 16SrRNA* gene (endogenous control) were designed and synthesized as “assay-by-design” from Applied Biosystems as listed in Table 1. Prior to proceeding with relative quantification, the cDNA template of 24 hour old biofilm samples with 10-fold serial dilutions was used to analyze the standard curves of both* algC* and* 16srRNA* gene. Real-time PCR was performed in 10 *μ*L reactions in 96-well PCR plates using 100 ng cDNA, 2X TaqMan gene expression master mix (Applied Biosystems, USA) and 20X* algC* assay mix (20X* 16SrRNA* assay mix in case of controls) in a 7500 fast real-time PCR system (Applied Biosystems, USA). The relative number of* algC* mRNA was determined using ΔΔCt-method (comparative threshold cycle) by normalizing Ct values of* algC* mRNA with that of* 16srRNA* in biofilm and planktonic samples at various time points from 3 to 96 hours. Relative quantification (RQ) represented in “fold over increase” was determined according to methods described elsewhere [32] and the formula used for calculation was RQ = 2^−ΔΔCt^. Cutoff Ct was kept 35, with an automatic threshold of 0.2 and baseline from cycle 3–15 with 95% confidence level. The 24-hour biofilm sample was set as the calibrator for this comparative gene expression analysis. Statistical comparison of RQ values (between biofilm and planktonic samples) was performed using Student's *t*-test and *P* value <0.05 was considered to be statistically significant.

### 2.6. Biofilm Development Assay

Biofilm development assays were performed to assess the pattern of biofilm formation of* A. baumannii* AIIMS 7 on polystyrene surface and for further correlation with gene expression plot over the same time line. Beginning at 3 h up to 96 h, quantitative biofilm assay was performed on 96-well microtitre plates, according to earlier methods [10] with appropriate modifications. Briefly,* A. baumannii* AIIMS 7 cultures (10^6^ CFU/mL, overnight grown) were inoculated onto polystyrene microtitre wells of a 96-well plate (Tarsons, India) and diluted initially with sterile distilled water (no nutrient broth) at a dilution of 1 : 40. After a static incubation of 3 hours at 37°C, the supernatants were aspirated and then sterile LB broth was added to the wells with a final dilution of 1 : 40 with sterile LB broth. For all time points (3–96 h), this was repeated in order to allow* A. baumannii* cells to attach onto polystyrene initially but not to grow and then to substitute it with addition of fresh Luria broth to facilitate adherent and micro-aggregated cells to develop biofilms. All plates were incubated at 37°C under static conditions for biofilm development, including negative controls (no cells, only nutrient medium) for each test samples in the plate. After appropriate incubation period(s), plates were taken out and nonadherent and dead cells were removed by brief sonication and aspiration carefully. The wells were washed thrice with sterile PBS, dried, and stained with 0.1% Gentian Violet (HiMedia, India). Excessive stain was removed by submerging the plate in a water trough and then dried in laminar air flow followed by addition 200 *μ*L of 100% ethanol for solubilizing the stained biofilm matrices. To measure the absorbance, plates were read in a Multi-Plate Reader (Molecular Devices, USA) at 570 nm before being shaken at 1020 rpm for 10 seconds. All biofilm development assays were performed in replicate of 12 and repeated. Absorbance values were termed as biofilm growth index after normalization with LB control, and values were plotted in Excel spreadsheets (Microsoft, USA) for further analysis.

### 2.7. Statistical Correlation of Biofilm Formation with* algC* Gene Expression

To find the concurrence of* algC* gene expression pattern with biofilm formation, RQ values (fold over increase in* algC* gene expression) in biofilm mode were correlated linearly with the biofilm indices at corresponding time points and* correlation coefficients* were determined using Excel spreadsheet (Microsoft, USA). Correlation statistics was restricted to three major stages, that is, initial attachment (3–9 hours), consolidation of the surface-attached micro-aggregation (12–24 hours), and maturation of biofilm (36–48 hours). 24-hour biofilm gene expression data (calibrator sample) was excluded from the correlation. *P* value of <0.05 was considered to be statistically significant.

### 2.8. Microscopic Examination of* A. baumannii* Biofilm Development

To localize the stages of biofilm growth over a temporal scale (3–96 h), biofilms were formed on sterile polystyrene culture dish, 55 mm × 15 mm (Tarsons, India), and incubated at 37°C under similar culture conditions as described above. After appropriate incubation periods, plates were taken out; supernatants were aspirated followed by washing three times with phosphate buffer saline for removal of nonadherent cells and fixed with 1 mL of methanol, air-dried, and observed without staining under a modular bright field microscope (Axioscope A1, Zeiss, Germany) with bright field settings at magnification of 100x.

### 2.9. Bioinformatics and Phylogenetic Analysis

Nucleotide and protein sequence comparisons were performed using BLASTn (National Center for Biotechnology Information, USA). Amino acid sequences were translated using Translate tool of the Expert Protein Analysis System (ExPASy, Swiss Institute of Bioinformatics). Protein sequences retrieved from NCBI were aligned using CLUSTALW [33] and phylogenetic tree was constructed with the help of analysis software MEGA v5.0 [34]. Bootstrapping was performed with 1,000 replicates for checking relative support for the branches in the phylogenetic tree. Comparison was restricted to sequences of close organisms, for example,* Pseudomonas aeruginosa*,* Pseudomonas putida*,* Pseudomonas syringae*, and previously predicted PMM sequence from whole genome sequence of* Acinetobacter baumannii* strain AYE,* Acinetobacter haemolyticus*, and* Acinetobacter calcoaceticus*. Higher eukaryote sequences, for example,* Oryctolagus cuniculus PGM* isoform 1 and PMM from* Homo sapiens*, were also included for comparison.

### 2.10. Cloning and Heterologous Expression

Upstream primer (alg_118F) and downstream primer (alg_1897R) were used in a PCR program with an additional 15 min final extension for producing poly-A tails in the PCR products to aid while TA cloning. Amplified* algC* was purified using gel purification kit (Bangalore GeNei, India) and ligated into pGEMT-Easy vector (Promega, USA) to produce resultant recombinant plasmid pGEalgCA7. The plasmid was transformed into chemically competent* E. coli* DH5*α*. Colonies were picked and direct PCR was performed to confirm presence of* algC* gene.

### 2.11. Assessment of AlgC (PMM/PGM) Protein Expression

Mid-log phase grown (with 100 mg/L ampicillin in Luria broth, under shaking conditions)* E. coli* DH5*α*-pGEalgCA7 and* E. coli* DH5*α*-pGEM-3Zf(+) at OD 600 = 0.8 were used to prepare whole cell extracts. The cell extracts were analyzed by SDS-PAGE (12.5%) and protein bands were documented in a gel documentation system (Alpha Innotech) after staining with 0.2% Coomassie brilliant blue (Himedia).

### 2.12. Assessment of Biofilm Augmentation

Biofilm augmentation was visualized by scanning electron microscopy. Briefly, overnight grown cells (3 × 10^9^ cells) were inoculated on (1 × 1 cm) sterile glass slides inside 12-well culture plates (Tarsons, India) and incubated at 37°C static. After 24 h of incubation, culture supernatant was removed; slides were immediately flooded with 2.5% glutaraldehyde in PBS and incubated at room temperature for 2 hours, followed by rinsing with sterile distilled water, and serially dehydrated with an ethanol gradient (25–100%), CO_2_-critical point dried and coated with platinum in an Auto Fine Coater (JFC-1600, JEOL, Japan). The slides were then observed in a scanning electron microscope (Vega, Tescan, USA) with 30 KV input voltage. For quantitative biofilm augmentation in the* algC* clones, biofilms were formed in microtitre plates and quantified as per methods described earlier [10].

### 2.13. Sequence Alignment and Building Model for PMM/PGM

PMM/PGM sequence of* A. baumannii* AIIMS 7 (AEC46864) was used as a target sequence in BLASTp program to identify possible template structures available in Protein Data Bank. The selected templates having good alignment score with the target sequence were aligned using CLUSTALW. Template protein structure 1K2Y.pdb (*P. aeruginosa* PMM/PGM S108A mutant), having maximum sequence alignment score (32%) with target sequence, was used as the final template to build homology model of PMM/PGM. Three-dimensional model of PMM/PGM was constructed using MODELLER 9 v7 [35] by considering 1K2Y.pdb as a template structure. The stability of the model was tested by using 10 ns molecular dynamics (MD) simulation.

### 2.14. Molecular Dynamics (MD) Simulation

MD simulations were performed on the homology model of PMM/PGM from* A. baumannii* AIIMS 7 in a HP workstation with the help of GROMACS 4.0.4 program using the GROMOS96 45a3 force field [36]. The structure was fully solvated with water (SPC) and system was neutralized with 9 Na^+^ ions. The solvated structure was minimized by steepest descent method for 1000 steps at 300 K temperature and constant pressure. The LINCS algorithm with 8.0 Å cutoffs was used for energy minimization of PMM/PGM whereas PME algorithm with 8.0 Å nonbonded cutoffs was utilized similarly as used in an earlier MD simulation study [37]. After equilibration period production MD was run for 10 ns at 300 K temperature, pressure, and constant volume ensemble. Solvent accessible surface (SA, Richards' surface) and molecular surface (MS, Connolly's surface) areas were calculated using CASTp analysis [38]. Structural comparison after molecular dynamics simulation of initial structure with final structure was done using PDBeFOLD software [39]. The MD simulation trajectory was then visualized using the VMD (visual molecular dynamics) package [40]. Three-dimensional images of enzyme PMM/PGM after MD simulation run were generated using the UCSF-Chimera tool [41] and Pymol (http://pymol.sourceforge.net/).

### 2.15. Nucleotide Sequence Accession

The GenBank accession number for* A. baumannii* AIIMS 7* algC* gene sequence is JF701279 and AEC46864 for the protein (PMM/PGM) sequence.

## 3. Results 

### 3.1. Identification of* algC* Gene in* A. baumannii* AIIMS 7

PCR with genomic DNA yielded a fragment of 1781 bp (Supplementary Figure S1 in the Supplementary Material available online at http://dx.doi.org/10.1155/2014/593546). PCR amplifications with plasmids were unsuccessful, confirming that the gene is not plasmid-borne. Reverse transcription PCR using total RNA showed amplification of internal coding regions 1 (1360 bp) and 2 (463 bp), which confirmed the active transcription of the* algC* ORF  (Supplementary Figure S2). DNA sequence of the gene was analyzed to predict regulatory regions such as promoter, ribosomal binding site, translation start, and end, which were identified and annotated (Supplementary Figure S3).

### 3.2. Elevated Levels of* algC* Gene Transcription during Biofilm Mode of Growth

**Figure 1:**
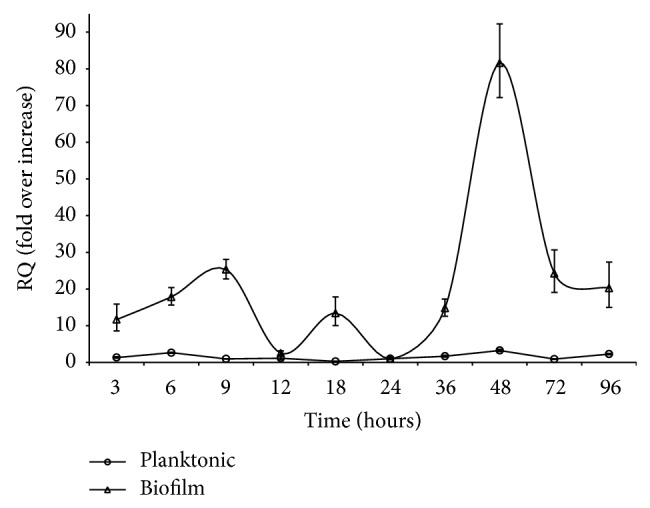
Comparison of* algC* gene expression pattern in biofilm and planktonic* A. baumannii* AIIMS 7. Real-time quantitative PCR results showing relative quantification of* algC* gene expression levels (calculated according to ΔΔCt-method and represented as “fold over increase”) at corresponding time points. Expression levels were normalized with indigenous control (*16SrRNA* gene expression) in both biofilm and planktonic cells (calibrator: 24-hour biofilm sample, fold over increase = 1).

Quantitative real-time PCR experiment showed significant variation in the threshold cycle (Ct) values in biofilm and planktonic mode of growth of* A. baumannii*, which described differential expressional patterns of the two modes of growth (Figure 1). Number of copies of* algC* mRNA were calculated using* 16SrRNA* gene as internal control at each time point (ΔCt). To evaluate the relative gene expression in biofilm and planktonic modes of growth over the temporal scale of 3 to 96 hours, the 24-hour biofilm sample was used as calibrator (ΔΔCt). Relative quantification (RQ) plot for gene expression at various time points from 3 to 96 hours showed low basal (almost linear) expression of* algC* in planktonic mode (maximum of 3.24-fold, 48 hours) whereas highly variable and elevated* algC* expression was seen in biofilm mode (maximum 81.59-fold, 48 hours) and displaying a definite pattern. The initial attachment stage (9 hours) and maturation stage (48 hours) of biofilm showed maximal expression of* algC*, which was in contrast and could be seen as steadily low expression at almost all time points in planktonic mode of growth (Figure 1). This observation affirms that the* algC* gene is highly up-regulated (at the transcription level) while the* A. baumannii* cells grow in a biofilm mode (i.e., attached to an abiotic surface) and follows a pattern as depicted (Figure 1); however in planktonic (free form, in a suspension, unattached to surface) the* algC* gene is transcribed at a basal rate.

### 3.3. Biofilm Growth Pattern Correlates with Quantitative* algC* Gene Expression

**Figure 2:**
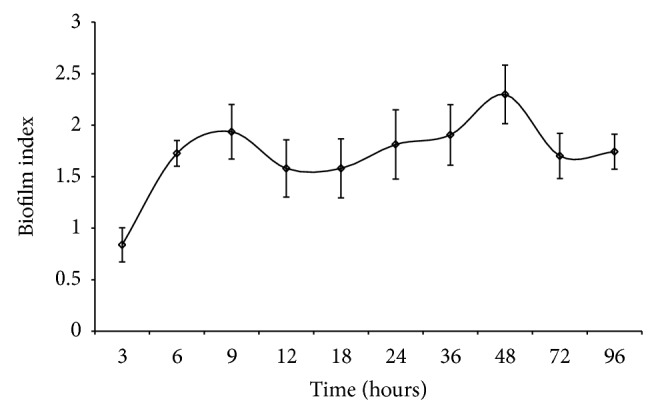
Pattern of biofilm growth of* A. baumannii* AIIMS 7. Graph showing corresponding biofilm indices of biofilms formed on polystyrene microtitre surface, calculated after normalization with control and plotted versus representative time points (three to 96 h).

Biofilm growth pattern* A. baumannii* on polystyrene surface can be seen in Figure 2, which can be explained in three distinct stages. First, an exponential rate of increase was observed at 3 to 9 hours, which defined the initial attachment and micro-colony aggregation stage (Figure 2). After 9 hour till 36 hours it showed steady level of increase in biofilm indices, indicating the consolidation stage of the attached micro-colonies and approaching maturation of biofilm. Third, maximum production of biofilm matrix could be seen at 36 to 48 hours, marking the maturation stage. Lastly, as like the plateau phase in the planktonic growth curve of* A. baumannii* AIIMS 7 (data not shown), the biofilm growth pattern also exhibited a steady state and persistent nature after maturation (after 48 hour till 96 hours). To find the association of* algC* gene expression pattern at transcription level (as revealed from real-time PCR analysis) with that of biofilm growth, correlation coefficients were determined using the RQ values in biofilm samples and biofilm indices at corresponding time points. High correlation was observed at all three major stages of biofilm. Correlation coefficients were 0.902, 0.925, and 0.983 (*P* < 0.05) corresponding to initial attachment, consolidation of the surface-attached micro-aggregation, and maturation of biofilm stages. Correlation was found to be maximum (0.983, *P* < 0.05) at stages where biofilms were mature. This indicated a strong possibility that transcription of* algC* could be in close association with biofilm formation, especially the maturation stage (36–48 hours) and also the initial attachment stage (3–9 hours).

### 3.4. Microscopic Visualization of Biofilm Development

**Figure 3:**
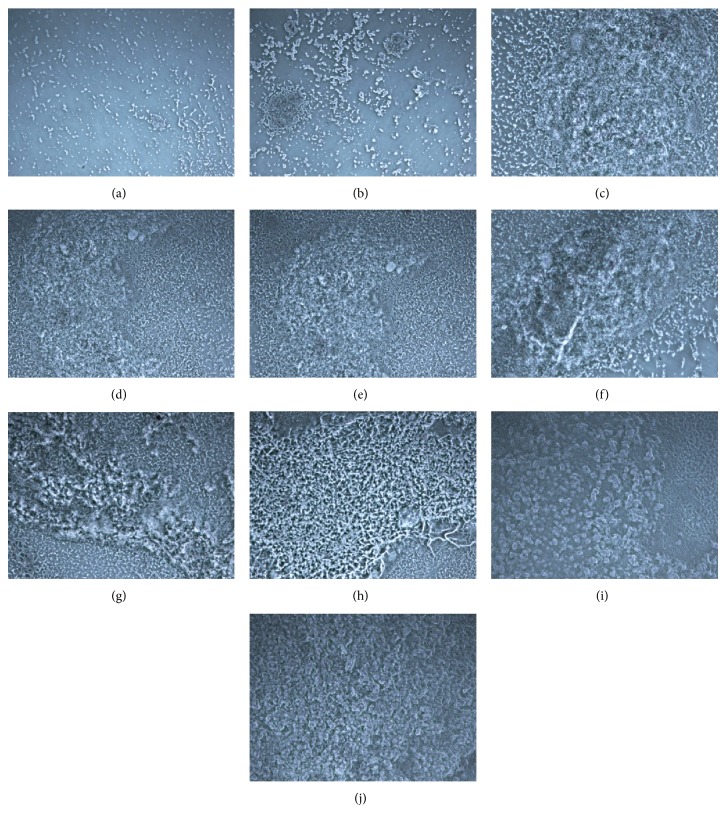
(a)–(j) Microscopic visualization of* A. baumannii* AIIMS 7 biofilms. Representative bright field micrographs depicting static biofilms formed on polystyrene microtitre surface at respective time points (3, 6, 9, 12, 18, 24, 36, 48, 72, 96 h) (magnification, 100x).

To visualize the growth pattern of biofilm, bright-field microscopy was performed on biofilms growing on polystyrene surface, which showed varied biofilm morphology at gradual time points (Figure 3) with a strong concurrence with quantitative evaluation of biofilm growth pattern (Figure 2, as described above). At initial attachment stages, planktonic cells were seen scantily aggregated (Figures 3(a) and 3(b)) but gradually the micro-aggregation on the surface increased, subsequently making completely attached micro-colonies at 9 h which could be seen clearly (Figure 3(c)). Further stages of growth (12, 18, and 24 h) showed consolidation of micro-colonies leading to stable structures of biofilm (Figures 3(d)–3(f)). At 36–48 h, fully mature biofilm communities of* A. baumannii* were seen, enriched with thick EPS matrices (Figures 3(g) and 3(h)), followed by appearance of robust three-dimensional biofilm structures, which were found persistent till 72 and 96 h (Figures 3(i) and 3(j)). Overall, visualization of biofilm growth at developing stages was in tandem with the patterns of biofilm growth as well as* algC* gene expression.

### 3.5. *In Silico* Analysis of Protein Reveals Highly Conserved Identity

**Figure 4:**
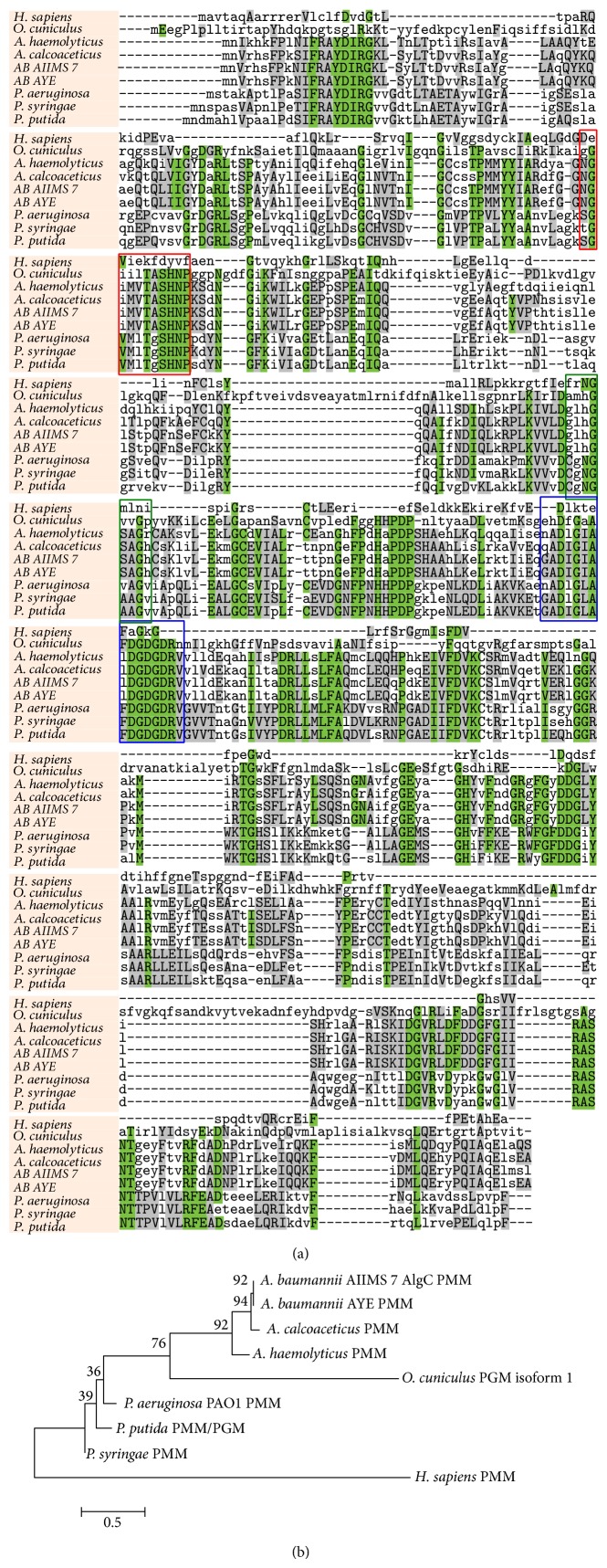
(a)-(b) Multiple sequence alignment and phylogenetic relationship of PMM/PGM. (a) CLUSTALW analyses of PMM/PGM protein sequences displaying representative color of box which highlights conserved regions, that is, active site (red), Mg^2+^ binding site (green), and substrate binding site (blue). (b) Bootstrap consensus tree for PMM/PGM enzymes, constructed by MEGA v5.0 using the neighbor-joining (NJ) method (based on 1,000 replicates). Numbers on the nodes are bootstrap values and the bar represents scale of estimated evolutionary distance (20 substitutions at any amino acid position per 100 amino acid positions).

To compare and evaluate the relatedness of* A. baumannii* AIIMS 7* algC* encoded PMM/PGM,* in silico* analyses were performed. BLASTn results showed 100% similarity with the predicted PMM/PGM sequence in whole genome sequences of* A. baumannii* available in NCBI database. Alignment results also displayed significant similarity with PMM/PGM nucleotide sequences of other genera. 472 amino acid residues could be theoretically translated in one frame (5′–3′) of the DNA sequence. BLASTp showed similar results as in BLASTn alignment. The molecular weight of the protein was predicted to be 52.94 kDa with isoelectric point (pI) of 5.67. CLUSTALW alignment with selected bacterial and eukaryotic PMM/PGM sequences showed conserved regions in the protein as shown in Figure 4(a) (active site, Mg^2+^ binding site, and sugar binding site marked as colored rectangles) in the protein as shown.  Figure 4(b) shows the phylogenetic tree indicating relatedness between PMM/PGM enzymes from pseudomonads and higher eukaryotes (rabbit and human). PMM/PGM sequence from* A. baumannii* had less than 25% identity with rabbit muscle PGM isoform 1, whereas it had a mere 10% identity with human PMM (Figure 4(b)).

### 3.6. Assessment of Protein

**Figure 5:**
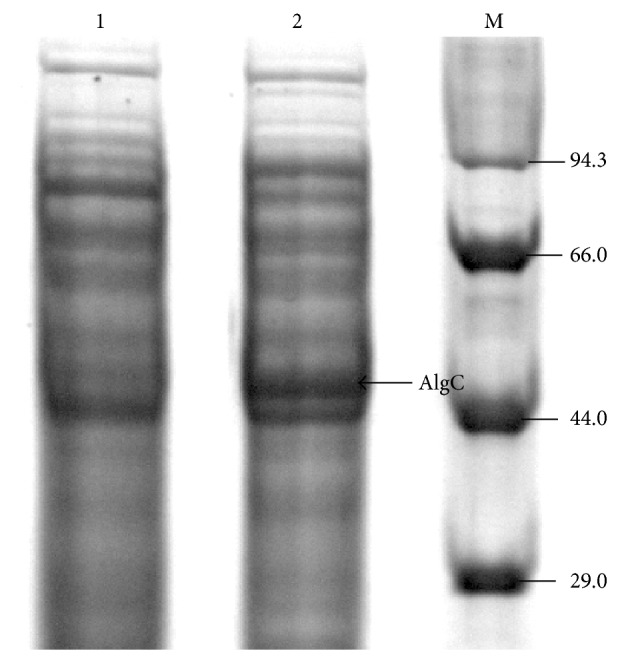
SDS-PAGE analysis of AlgC (PMM/PGM) protein. Lane 1: whole cell extract of control* E. coli* DH5*α* (lacking* algC* gene); lane 2: whole cell extract of* E. coli* DH5*α* with* algC* gene showing the AlgC protein band** ~**53 kDa; lane M: standard protein molecular weight marker (kDa).

To demonstrate the synthesis of PMM/PGM protein, whole cell extracts of cloned* E. coli* DH5*α* with resultant recombinant plasmid pGEalgCA7 cells were used in SDS-PAGE analysis, which showed a clearly overexpressed protein of ~53 kDa suggesting the PMM/PGM protein in question (Figure 5). Control* E. coli* DH5*α* (with empty vector pGEM-3Zf+ lacking an* algC* gene) did not show any PMM/PGM protein band.

### 3.7. Biofilm Augmentation by* algC* after 24 h

**Figure 6:**
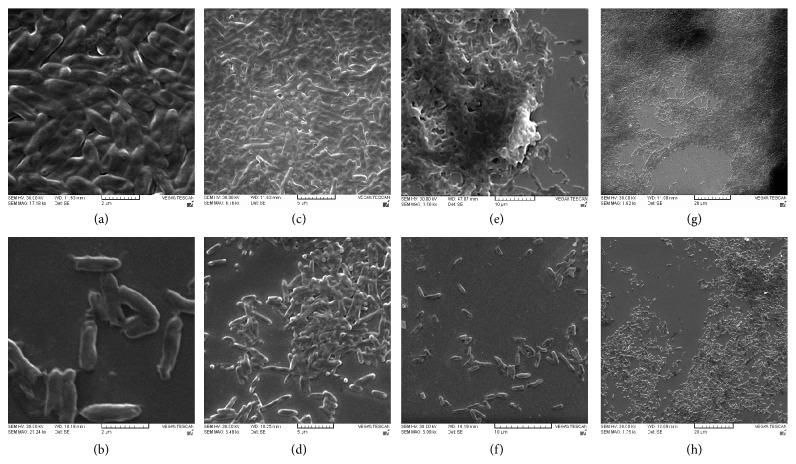
(a)–(h) Visualization of biofilm augmentation by scanning electron microscopy. Electron micrographs (a, c, e, g) showing biofilms formed by* algC* clones after 24 h, at a gradient of magnifications (Bar = 2 *μ*m, 5 *μ*m, 10 *μ*m, and 20 *μ*m; absolute magnifications indicated as Kx in the very picture) as observed under a scanning electron microscope.* algC* clones can be seen clearly producing dense and robust biofilm as suggested by the thickness and integrity of the biofilm matrices, compared to the control biofilms by* E*.* coli* DH5*α* (lacking* A. baumannii algC* gene) at corresponding comparable magnifications (b, d, f, h).

Electron micrographs of biofilm formed by the* algC* clones (Figure 6) showed significant increase in biofilm formation compared to controls, probably indicative of the overexpression of the PMM/PGM protein and the resultant exopolysaccharides. Thicker biofilms could be seen in the* algC* clones having clear dense matrices (Figures 6(a), 6(c), and 6(e)) with a intracellular cementing material clearly visible (Figures 6(a) and 6(e)) indicative of the biofilm EPS. Overall SEM analysis at a gradient of magnifications and concurrent comparisons with control clones (lacking functional copy of* algC*) suggested substantially that there is significant facilitation of the overall biofilm formation, emphasizing the enrichment of the biofilm matrices, which could be due to the cohesive activity of EPS including alginate and LPS cores. The quantitative biofilm assay concurred with the microscopic analysis, with augmentation of biofilm up to 3.87-fold in the* algC* clones compared to the control cells lacking* algC* gene (*P* < 0.02, data not shown).

### 3.8. Molecular Dynamics Simulation Study of PMM/PGM

**Figure 7:**
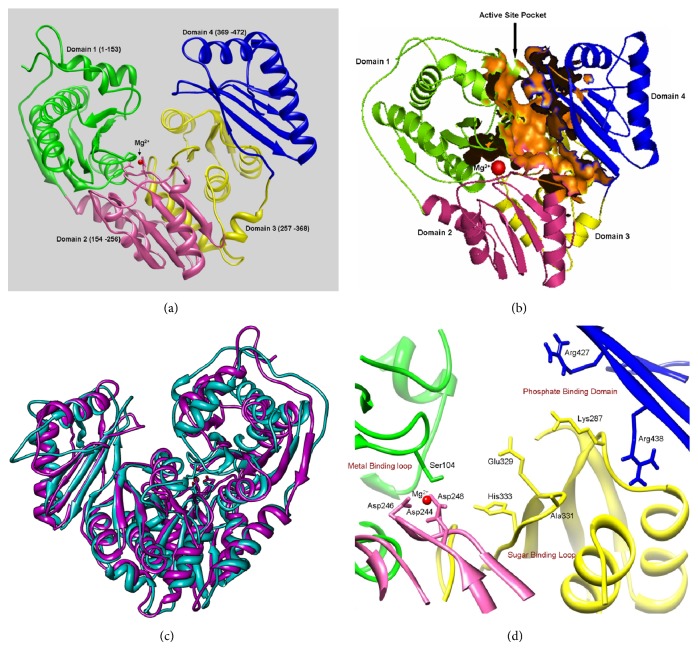
(a)–(d) Molecular modeling of PMM/PGM from* A. baumannii* AIIMS 7. (a) Four sequential domains of enzyme PMM/PGM after MD simulation; (b) central spherical cavity showing solvent accessible surface area (orange). (c) Superimposed structures before MD (cyan) and after MD simulation (magenta); (d) Mg^2+^ interacting residues of PMM/PGM enzyme.

To predict the functional association between PMM/PGM enzyme (encoded by* algC* gene) resulting in alginate/LPS core mediated biofilm formation in* A. baumannii*, molecular dynamics (MD) simulations up to 10 ns were carried out on homology model of PMM/PGM of* A. baumannii* AIIMS 7 using GROMACS v4.0.4 program. The generated PMM/PGM model of* A. baumannii* AIIMS 7 contained four domains of equal size arranged in “heart shaped” manner (Figure 7(a)) which form a compact structure, similar to* P. aeruginosa* [42]. The polypeptide chain proceeds through each domain sequentially (Figure 7(a)) forming heart shaped geometry. Sequence alignments (Supplementary Figure S4) and model building study showed that the active site was located at the centre of the two domains (domains 1 and 2) in a deep cleft formed by Ser104, Asp244, Asp246, and Asp248. PMM/PGM sequence of* A. baumannii* AIIMS 7 showed conserved sequence motif in domain 3 from residues 327-331 (GEYAGH) which would act as a sugar binding site. A cluster of positively charged conserved residues found in domain 3 (Lys287) and domain 4 (Arg427, Arg438) could be involved in phosphate binding. The cleft showed solvent accessible surface (SA, Richards' surface) and molecular surface (MS, Connolly's surface) areas as calculated by alpha shape method. The enzyme showed a total cavity of 3641.5 Å and spherical central cavity of 1878.13 Å where substrate could bind as shown in Figure 7(b). To compare the 3D structures obtained before and after MD simulations, structural superpositions were made using PDBeFOLD, results of which showed root mean square deviation (RMSD) of two aligned structures within the range of 2.51 Å (Figure 7(c)). Metal ion Mg^2+^ interacts with Asp244, Asp246, Asp248, and Ser104 residues (Figure 7(d); Supplementary Table S1). The metal ion Mg^2+^ is required for enzymatic activity of PMM/PGM of* A. baumannii* AIIMS 7 (Figure 7(d)), rather than Mn^2+^ and Zn^2+^. Specific interactions between metal ion (Mg^2+^) and protein, water-mediated H-bonds (so-called water bridges), and hydrophobic (Lennard-Jones) interactions have been identified and are listed (Supplementary Table S1).

### 3.9. Stability of Phosphomannomutase during Simulation

**Figure 8:**
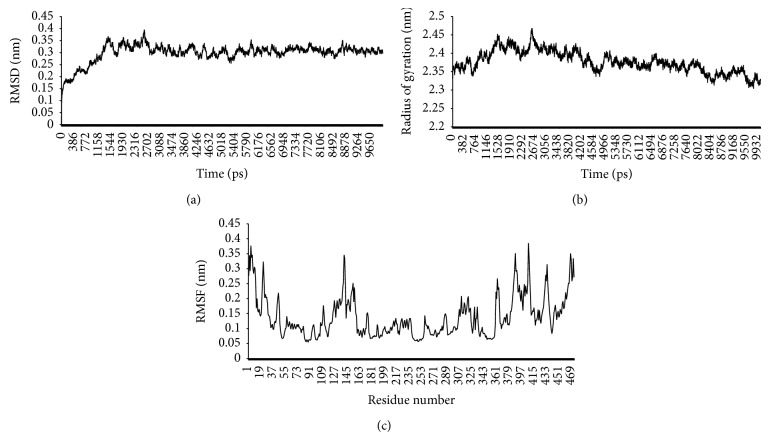
(a)–(c) Results from 10 ns molecular dynamics simulation performed on PMM/PGM model. (a) Root mean square deviation (RMSD); (b) radius of gyration; (c) root mean square fluctuation (RMSF).

To verify the simulation stability and to measure structure and dynamics of PMM/PGM model of* A. baumannii* AIIMS 7, standard structural parameters like RMSD, root mean square fluctuation (RMSF), and radius of gyration (RG) were calculated and represented in Figures 8(a)–8(c). During molecular dynamics simulation period of 10 ns, the average RMSD value was 0.3 nm as depicted (Figure 8(a)). Steady RMSD result showed for the atoms in the four domains of PMM/PGM model indicated that the catalytic activity is relatively stable during the simulation. The result for radius of gyration (RG) also indicated the stability of model over the whole simulation period (Figure 8(b)). Minor fluctuations in C-alpha atoms (RMSF) of helical and sheet structural elements could be seen whereas loop region showed variability to some extent (Figure 8(c)). Overall, these results of RMSD, RMSF, and RG strongly indicated the stability of system over the entire simulation period.

## 4. Discussion

Biofilm formation substantially aids to the spectrum of multidrug resistance displayed by* A. baumannii* and is often attributed as the major cause for antibiotic treatment failure [3, 5]. The process of bacterial biofilm development is believed to be a complex interplay of the biofilm EPS matrix components, that is, a plethora of proteins and extracellular macromolecules, for example, DNA and polysaccharides. The synthesis of specific exopolysaccharides such as alginate and LPS core has been described in various bacterial systems [25, 43, 44], in particular* P. aeruginosa*, with its contribution in biofilm formation [20, 22]. However,* algC* gene function and its expression pattern have not been elucidated in* A. baumannii* in order to understand the genetic basis of its association with biofilm formation. Herein, an initial genetic characterization of the* algC* gene and its association with biofilm formation is reported in the MDR strain AIIMS 7 of* A. baumannii*, which depicts differential expression of* algC* gene during the biofilm development. Besides, MD simulation was performed on the three-dimensional (3D) structure of the gene product (enzyme PMM/PGM) to compare with that of* P. aeruginosa*, followed by its cloning, heterologous expression, and phylogenetic analysis. Finally, biofilm augmentation by* algC* gene was evaluated quantitatively and confirmed by SEM.

Analysis of the* algC* gene expression pattern in the temporal scale (up to 96 hours) reveals that surface-attached bacteria have significantly higher rate of transcription levels than nonadherent or shaking cultures. The attachment or “surface sensing” could be the trigger for the activation of* algC *promoter, as a result of which the* algC* expression could be seen consistently higher (maximum 81.59-fold, *P* < 0.05) in biofilm forming* A. baumannii* cultures than the planktonic counterparts (maximum 3.24-fold) as revealed by real time PCR analysis (Figure 1). This observation is in accordance with an earlier study in* P. aeruginosa* [45] which shows* algC* reporter gene activity in continuous biofilm culture cells to be 19-fold increased than planktonic cells. Earlier, Davies et al. [46] have shown that expression of* P. aeruginosa algC* is upregulated after initial attachment of cells to teflon or glass substrata, where cells that failed to upregulate alginate biosynthesis typically detached from the surface. As reviewed earlier by Gacesa [47], it is presumed that exopolysaccharide alginate plays a role in consolidation of the biofilm rather than in the initial adhesion event and hence higher rate of* algC* transcription was not a prerequisite to surface attachment by* P. aeruginosa*, indicating alginate biosynthesis not necessary for its attachment to glass surface. In contrast, our data in support of the relative quantification of* algC* transcription by real-time PCR and biofilm augmentation by* algC* as visualized by SEM is indicative of the possible involvement of* algC* expression in probably both the events, that is, initial attachment and consolidation of biofilms formed by* A. baumannii*. Furthermore, the upregulation of* algC* transcription at the maturation stages (36–48 hours) explains that it is strongly associated with maturation of biofilms, as the correlation of* algC* expression with biofilm formation during the very stage was significantly higher than the initial events (0.983 compared to 0.902, *P* < 0.05). During planktonic mode of growth, low levels of* algC* transcription were observed as compared to biofilm forming* A. baumannii*, which should be ideally due to lack of surface attachment (less promoter activity) and variations in the oxygen tension in shaking and static cultures. Regulation of the* algC* gene could be dependent on diverse parameters as seen in* P. aeruginosa*, where it was shown to be in coordination with the* algC* promoter activity [23]. Besides, according to other studies, variations in oxygen tension [48], nitrogen limitation [49], and ethanol induced membrane perturbation [50] can also influence the alginate production in* P. aeruginosa*. In* A. baumannii*, we demonstrate differential* algC* transcription to be dependent upon abiotic (polystyrene) surface attachment and thus, further investigation would be required to portray a comprehensive profile of* algC* genetic regulation.

The 3D structure of PMM/PGM bifunctional enzyme as determined by homology modeling, which is the gene product of* algC* from* A. baumannii* strain AIIMS 7, shows 32% overall similarity. Furthermore, high sequence identity of the catalytic domain was observed with that of experimentally determined structure of PMM/PGM of* P. aeruginosa* [42]. Each of the four domains contained residues essential for catalysis and/or substrate recognition. While the catalytic phosphoserine residue (Ser104) of domain 1 could be useful to transfer phosphoryl group to and from the bisphosphorylated reaction intermediate, domain 2 was found to contain a metal binding loop (Asp244, Asp246, Asp248) that could coordinate the Mg^2+^ ion required for the PMM/PGM activity (Figure 7(d)). Domain 3 of PMM/PGM of* A. baumannii* also contains sugar binding loop (Glu327, Ala329, His331) similar to that in PMM/PGM of* P. aeruginosa*. This sugar binding loop has key residues which could enable the enzyme to recognize the two different binding orientations of its 1- and 6-phospho sugar substrates. Domains 3 and 4 provide most of the residues that create a phosphate binding site (Lys287 of domain 3 and Arg427, Arg438 of domain 4) that determines the orientation of the incoming phosphor-sugar substrates. In context with previous studies in* P. aeruginosa* PMM/PGM [27, 51], our investigation predicts that PMM/PGM of* A. baumannii* requires Mg^2+^ and activator glucose 1,6-biphosphate for its catalytic activity. Other physical properties of the protein analyzed* in silico* were also found to be similar to those of PMM/PGM enzyme from* P. aeruginosa* [42]. This indicated a similar function of the enzyme, that is, a bifunctional role which would be responsible for conversion of mannose-6-phosphate to mannose-1-phosphate or glucose-6-phosphate to glucose-1-phosphate as in* P. aeruginosa* [28].

The active site residues predicted in the PMM/PGM enzyme 3D structure were determined by multiple sequence alignments and, however, needs to be validated by experimental analysis. In context of sugar binding loop, PMM/PGM of* A. baumannii* may also have equal specificity for glucose as well as mannose, because mannose and glucose are epimers at C-2. The active site residues of characterized PMM/PGM complexes are known to change conformation based on the identity of the bound ligand and/or change the type of contact in which they participate [28, 52]. However, a direct structural characterization of multiple enzyme-substrate complexes would likely be necessary for a full understanding of substrate specificity in this enzyme superfamily. Nevertheless, the MD simulation results in this research reveal several regions of the PMM/PGM enzyme to be highly conserved and clearly important for function in all organisms, supporting the proposal that all of these enzymes use a common mechanism. The* algC* gene of* A. baumannii* AIIMS 7 characterized in this study is an ortholog of* algC* gene from* P. aeruginosa*. The PMM/PGM protein plays a central role in the production of three* P. aeruginosa* virulence-associated traits, alginate, rhamnolipids, and LPS, although the production of these compounds is subjected to a completely different genetic and environmental regulation [23, 45]. The PGM activity of the protein is involved in synthesis of the LPS core of biofilms, while PMM activity is responsible for alginate biosynthesis. Both of these (PMM/PGM) enzymatic functions either singly or in combination could be attributed for the biofilm formation in* A. baumannii* AIIMS 7 and augmentation of biofilms in heterologous clones as observed by SEM. It is also conceivable, therefore, that the cloned and overexpressed PGM enzyme (of* A. baumannii* AIIMS 7) might have used up the machinery for cellular LPS core biosynthesis in* E. coli* [25] and could be the possible mechanism behind the overall augmentation of biofilms in the heterologous* algC* clones (*E. coli* DH5*α*, a relatively weak biofilm former than* A. baumannii*) as evident from the SEM image analysis. Nevertheless, due to the central role of PMM/PGM in biosynthetic pathways, the enzyme has potential to serve as a novel marker for* A. baumannii* infection or as pharmaceutical targets for inhibition. Such inhibitors would be specific and not affect the essential homolog in human, due to lack of conservation in active site, sugar, and metal binding site.

The mechanism of PMM/PGM is believed to be closely parallel to that of related proteins from other pseudomonads including close relatives like* P. aeruginosa*. Our observations on the similarity of the protein sequences and 3D models indicate that the PMM/PGM protein of* A. baumannii* AIIMS 7 could have a similar biological role in biofilms as seen in* P. aeruginosa*. Therefore, the dual roles of PMM/PGM in alginate and LPS biosynthesis enhancing the biofilm formation in* A. baumannii* propose the enzyme as an attractive target for inhibitor design.

## 5. Conclusion

This study presents an initial genetic characterization of* algC* gene in an MDR strain* A. baumannii* AIIMS 7 which demonstrates a quantitative differential expression pattern in biofilm cells compared to planktonic counterparts cells over a temporal scale of 96 h. Molecular dynamics (MD) simulation studies indicate that the 3D structure of enzyme (PMM/PGM) has functions similar to* P. aeruginosa* which might result in significant biofilm augmentation in heterologous* algC* clones, as evident from SEM analysis. These experimental lines of evidence collectively suggest that the differential* algC* gene transcription in* A. baumannii* strongly correlates with the initial attachment (9 h) and maturation stages (36 h) during the biofilm development, however, may not be the only attribute contributing to biofilm formation and could be associated with other factors. The PMM/PGM enzyme can be a potential drug target for biofilm-associated infections of* A. baumannii* as revealed by the 3D structural simulation studies.

## Supplementary Material

We performed molecular dynamic simulation on the 3-D model of the *Acinetobacter baumannii* AIIMS 7 PMM/PGM enzyme, which revealed data on the specific chemical interactions between metal ion (Mg^2+^) and the PMM/PGM protein. Moreover, the water-mediated Hydrogen bonds (water bridges) and hydrophobic interactions (Lennard-Jones) were also identified, as mentioned in the **Supplementary Table S1**. Regarding the genetic characterization of the *algC* gene of the MDR strain *A. baumannii* AIIMS 7, amplification of the *algC* gene from genomic DNA was performed using specifically designed primers (118_F / 1897_R, Table 1) and documented after anlaysis by agarose gel electrophoresis as demonstrated in **supplementary Figure S1**. Similarly, to verify the transcription of the *algC* gene, total RNA was isolated and two internal regions of *algC* ORF (1360 bp and 463 bp) were amplified from the cDNA using primer pair 363F / CD1701R, and internal nested primer pair 1260 nest1F / CD1701R respectively (Table 1); as illustrated in the **supplementary Figure S2**. After sequencing the 1781 bp *algC* gene, the *in silico* tool (Neural Network Promoter Prediction program http://www.fruitfly.org/seq_tools/promoter.html) was used to predict the promoter region of the gene, results of which has been depicted in the **supplementary Figure S3**. Regulatory elements of the *algC* gene such as promoter, ribosomal binding site (RBS), transcription start site, the -10 and -35 regions of the promoter, translation start and end, could be identified and annotated. **supplementary Figure S4** shows the CLUSTALW alignment of target PMM/PGM sequence (AEC46864) from A. *baumannii* AIIMS 7 with the template sequence (1K2Y) from *P. aeruginosa* PMM/PGM S108A mutant. This alignment was very crucial for building the 3-D model of the PMM/PGM protein from *A. baumannii* AIIMS 7 and subsequent Molecular Dynamics simulation studies. The alignment of these two protein sequences revealed the respective metal binding residues, sugar binding residues and phosphate binding residues as illustrated using colored highlights.

## Figures and Tables

**Table 1:** Oligos/probes used for PCR, RT-PCR, and real-time PCR analysis.

Gene	Primer	5′-3′ Nucleotide sequence
*algC *	118_F	TTAGAACCGGGTGAGCGTTTAGCA
	1897_R	AGATGCTGATCTTGTGGCATTGCG

*al* *gC* ^*ψ*^	ALGC1_F	GCTGTGAAGTCATTGCTTTACGT
	ALGC1_R	TGAAGGGTCTGGTGCATGATC
	ALGC1_MFAM	6FAM-CAAATGGTGAGTTCCC-TAMRA

*algC cDNA int_region 1 *	CD363F	CCGTGCTTACGATATTAGAGGCAA
	CD1701R	AATTTGCGGATAACGCTCTTGC

*algC cDNA int_region 2 *	1260nest1	CTTCCTCCGCGCGTATTTATC
	CD1701R	AATTTGCGGATAACGCTCTTGC

*16SrRNA *	16B-F	TGGCTCAGATTGAACGCTGGCGGC
	16B-R	TACCTTGTTACGACTTCACCCCA

16*SrRNA* ^*ψ*^	16SRR_F	TGTCGTGAGATGTTGGGTTAAGTC
	16SRR_R	CCGAAATGCTGGCAAGTAAGGA
	16SRR_MFAM	6FAM-ACGAGCGCAACCCTTT-TAMRA

^*ψ*^Primers for real-time PCR assays.
