# Supplementary material for: Characterization of the algC Gene Expression Pattern in the Multidrug Resistant Acinetobacter baumannii AIIMS 7 and Correlation with Biofilm Development on Abiotic Surface
Source: ScientificWorldJournal. 2014 Dec 3;2014:593546. doi: 10.1155/2014/593546 (PMC4269089; doi:10.1155/2014/593546)
Supplement: Supplementary file 1 — We performed molecular dynamic simulation on the 3-D model of the Acinetobacter baumannii AIIMS 7 PMM/PGM enzyme, which revealed data on the specific chemical interactions between metal ion (Mg2+) and the PMM/PGM protein. Moreover, the water-mediated Hydrogen bonds (water bridges) and hydrophobic interactions (Lennard-Jones) were also identified, as mentioned in the Supplementary Table S1. Regarding the genetic characterization of the algC gene of the MDR strain A. baumannii AIIMS 7, amplification of the algC gene from genomic DNA was performed using specifically designed primers (118_F / 1897_R, Table 1) and documented after anlaysis by agarose gel electrophoresis as demonstrated in supplementary Figure S1. Similarly, to verify the transcription of the algC gene, total RNA was isolated and two internal regions of algC ORF (1360 bp and 463 bp) were amplified from the cDNA using primer pair 363F / CD1701R, and internal nested primer pair 1260 nest1F / CD1701R respectively (Table 1); as illustrated in the supplementary Figure S2. After sequencing the 1781 bp algC gene, the in silico tool (Neural Network Promoter Prediction program http://www.fruitfly.org/seq_tools/promoter.html) was used to predict the promoter region of the gene, results of which has been depicted in the supplementary Figure S3. Regulatory elements of the algC gene such as promoter, ribosomal binding site (RBS), transcription start site, the -10 and -35 regions of the promoter, translation start and end, could be identified and annotated. supplementary Figure S4 shows the CLUSTALW alignment of target PMM/PGM sequence (AEC46864) from A. baumannii AIIMS 7 with the template sequence (1K2Y) from P. aeruginosa PMM/PGM S108A mutant. This alignment was very crucial for building the 3-D model of the PMM/PGM protein from A. baumannii AIIMS 7 and subsequent Molecular Dynamics simulation studies. The alignment of these two protein sequences revealed the respective metal binding residues, sugar binding residues [file 593546.f1.pdf]

Legends for Supplementary Tables and Figures

Table S1 Interactions of metal ion (Mg^{2+}) with *A. baumannii* AIIMS 7 PMM residues

Fig. S1 Amplification of *algC* from genome of *A. baumannii* AIIMS 7

Lane M: Molecular weight marker (in kb); lane 1: *algC* gene (1781 bp) amplified from genomic DNA; Lane 2: Negative PCR control (DNase, RNase free water)

Fig.S2 Amplification of internal coding regions of cDNA of *algC* by RT-PCR assay

Lane M: Molecular weight marker (in kb), lane 1: coding region 1 (463 bp); lane2: coding region 2 (1360 bp) amplified from cDNA; Lane 3: Negative PCR control (total RNA used as template for direct PCR)

Fig. S3 Promoter region of *algC* gene

algC transcription initiation site and the direction of transcription are indicated by the large, bold type and the black curved arrow, respectively. The predicted matching -10 (GATAGA) and -35 (TTGAATA) promoter regions and a putative RBS (highlighted in grey) are also shown. The black horizontal arrow identifies the *algC* initiation codon.

Fig. S4 CLUSTALW alignment of target and template sequences used for 3-D model building

Alignment of target PMM/PGM sequence(AEC46864) from *A. baumannii* AIIMS 7 with template sequence (1K2Y) from *P. aeruginosa* PMM/PGM S108A mutant. Respective colored regions show: metal binding residues, Ser104(domain 1, Green), Asp244, Asp246, Asp248 (domain 2, Pink);sugar binding residues, Glu327, Ala329, His331 (domain 3, Yellow); phosphate binding residues: Lys287 (domain 3, yellow), Arg 427, Arg 438 (domain 4, Blue)

SUPLEMENTARY TABLES & FIGURES

Suppl. Table S1 Interactions of metal ion (Mg^{2+}) with *A. baumannii* AIIMS 7 PMM residues

**S.No**	**Residues**	**Distance in Å**
1	SER 104 OG ---- Mg	2.006
2	ASP 244 OD2 ----Mg	2.214
3	ASP 244 OD1 ----Mg	2.032
4	ASP 248 OD1 ----Mg	2.060
5	ASP 248 OD2 ----Mg	2.138
6	ASP 246 OD2 ----Mg	2.057
7	ASP 246 OD1 ----Mg	2.051

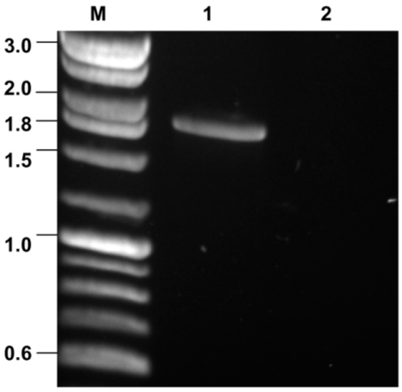

Suppl. Fig. S1 Amplification of *algC* from genome of *A. baumannii* AIIMS 7

Lane M: Molecular weight marker (in kb); lane 1: *algC* gene (1781 bp) amplified from genomic DNA; Lane 2: Negative PCR control (DNase, RNase free water

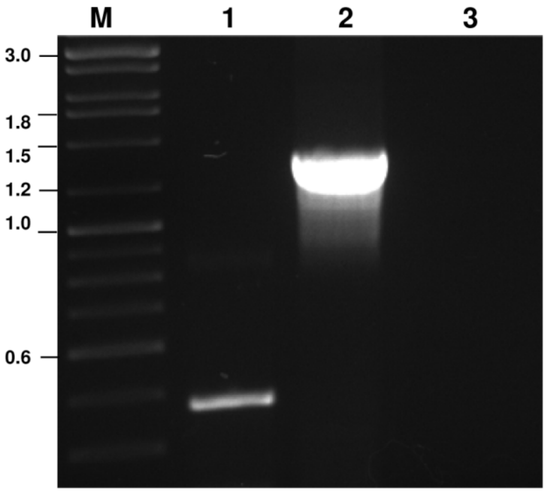

Suppl. Fig.S2 Amplification of internal coding regions of cDNA of *algC* by RT-PCR assay

Lane M: Molecular weight marker (in kb), lane 1: coding region 1 (463 bp); lane2: coding region 2 (1360 bp) amplified from cDNA; Lane 3: Negative PCR control (total RNA used as template for direct PCR)

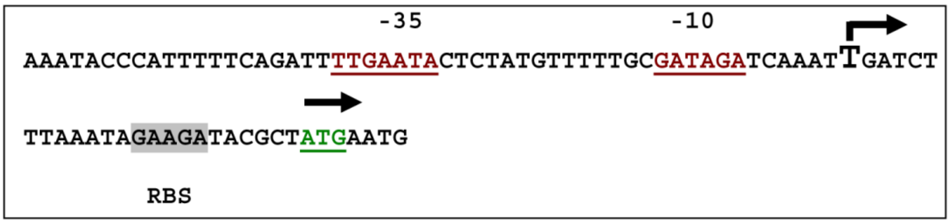

Suppl. Fig. S3 Promoter region of *algC* gene

algC transcription initiation site and the direction of transcription are indicated by the large, bold type and the black curved arrow, respectively. The predicted matching -10 (GATAGA) and -35 (TTGAATA) promoter regions and a putative RBS (highlighted in grey) are also shown. The black horizontal arrow identifies the *algC* initiation codon.

```
CLUSTAL 2.0.11 multiple sequence alignment


gi|330858329|gb|AEC46864.1|      --MNVRHSFPKNIFRAYDIRGKLS-YLTTDVVRSIAYGLAQQYKQAEQTQ 47
1K2Y_X|PDBID|CHAIN|SEQUENCE      MSTAKAPTLPASIFRAYDIRGVVGDTLTAETAYWIGRAIGSESLARGEPC 50
                                        ::* .********* :.   **::..  *. .:..:     :.

gi|330858329|gb|AEC46864.1|      LIIGYDARLTSPAYAHLIEEILVEQGLNVTNIGCCSTPMMYYIAREFGG- 96
1K2Y_X|PDBID|CHAIN|SEQUENCE      VAVGRDGRLSGPELVKQLIQGLVDCGCQVSDVGMVPTPVLYYAANVLEGK 100
                                 : :* *.**:.*  .: : : **: * :*:::*  .**::** *. : *

gi|330858329|gb|AEC46864.1|      NGIMVTASHNPKSDNGIKWILRGEPPSPEMIQQVGEFAQTYVPTHTISLL 146
1K2Y_X|PDBID|CHAIN|SEQUENCE      SGVMLTGAHNPPDYNGFKIVVAGETLANEQIQALRERIEKNDLASGVGSV 150
                                 .*:*:*.:*** . **:* :: **. : * ** : *  :.   :  :. :

gi|330858329|gb|AEC46864.1|      ELSTPQFNSEFCKKYQQAIFNDIQLKRPLKVVLDGLHGSAGHCSKLVLEK 196
1K2Y_X|PDBID|CHAIN|SEQUENCE      EQ------VDILPRYFKQIRDDIAMAKPMKVVVDCGNGVAGVIAPQLIEA 194
                                 *        ::  :* : * :** : :*:***:*  :* **  :  ::*

gi|330858329|gb|AEC46864.1|      MGCEVIALRTTPNGEFPDHAPDPSHAAHLKELRKTIIEQGADIGIALDGD 246
1K2Y_X|PDBID|CHAIN|SEQUENCE      LGCSVIPLYCEVDGNFPNHHPDPGKPENLKDLIAKVKAENADLGLAFDGD 244
                                 :**.**.*    :*:**:* ***.:. :**:*  .:  :.**:*:*:***

gi|330858329|gb|AEC46864.1|      GDRVVLLDEKANILTADRLLSLFAQMCLEQQPDKEIVFDVKCSLMVQRTV 296
1K2Y_X|PDBID|CHAIN|SEQUENCE      GDRVGVVTNTGTIIYPDRLLMLFAKDVVSRNPGADIIFDVKCTRRLIALI 294
                                 **** :: :...*: .**** ***:  :.::*. :*:*****:  :   :

gi|330858329|gb|AEC46864.1|      ERLGGKPKMIRTGSSFLRAYLSQSNGNAIFGGEYAGHYVFNDGRGFGYDD 346
1K2Y_X|PDBID|CHAIN|SEQUENCE      SGYGGRPVMWKTGHSLIKKKMKETG--ALLAGEMSGHVFFKER-WFGFDD 341
                                 .  **:* * :** *:::  :.::.  *::.** :** .*::   **:**

gi|330858329|gb|AEC46864.1|      GLYAALRVMEYFTESSATTISDLFSNYPERCCTEDTYIGTHQSDPKHVLQ 396
1K2Y_X|PDBID|CHAIN|SEQUENCE      GIYSAARLLEILSQDQRDSE-HVFSAFPSDISTPEINITVTEDSKFAIIE 390
                                 *:*:* *::* :::..  :  .:** :*.  .* :  * . :..   :::

gi|330858329|gb|AEC46864.1|      DIEILSHRLGARISKIDGVRLDFDDGFGIIRASNTGEYFTVRFDADNPLR 446
1K2Y_X|PDBID|CHAIN|SEQUENCE      ALQRDAQWGEGNITTLDGVRVDYPKGWGLVRASNTTPVLVLRFEADTEEE 440
                                  ::  ::   ..*:.:****:*: .*:*::*****   :.:**:**.  .

gi|330858329|gb|AEC46864.1|      LKEIQQKFIDMLQERYPQIAQELMSL 472
1K2Y_X|PDBID|CHAIN|SEQUENCE      LERIKTVFRNQLKAVDSSLPVPF--- 463
                                 *:.*:  * : *:   ..:.  :
```

Suppl. Fig. S4 CLUSTALW alignment of target and template sequences used for 3-D model building

Alignment of target PMM/PGM sequence(AEC46864) from *A. baumannii* AIIMS 7 with template sequence (1K2Y) from *P. aeruginosa* PMM/PGM S108A mutant. Respective colored regions show: metal binding residues, Ser104(domain 1, Green), Asp244, Asp246, Asp248 (domain 2, Pink);sugar binding residues, Glu327, Ala329, His331 (domain 3, Yellow); phosphate binding residues: Lys287 (domain 3, yellow), Arg 427, Arg 438 (domain 4, Blue)
